# Specialty career preferences among final year medical students at Makerere University College of health sciences, Uganda: a mixed methods study

**DOI:** 10.1186/s12909-021-02630-x

**Published:** 2021-04-16

**Authors:** Job Kuteesa, Victor Musiime, Ian G. Munabi, Aloysius G. Mubuuke, Robert Opoka, David Mukunya, Sarah Kiguli

**Affiliations:** 1 Department of Surgery, School of Medicine, Makerere University College of Health Sciences, Kampala, Uganda; 2 Research Department, Joint Clinical Research Centre, Kampala, Uganda; 3 Department of Pediatrics, School of Medicine, Makerere University College of Health Sciences, Kampala, Uganda; 4 Department of Human Anatomy, School of Biomedical Sciences, Makerere University College of Health Sciences, Kampala, Uganda; 5 Department of Radiology, School of Medicine, Makerere University College of Health Sciences, Kampala, Uganda; 6 Sanyu Africa Research Institute, Mbale, Uganda

**Keywords:** Career, Medical, Preferences, Specialty, Student

## Abstract

**Background:**

Uganda has an imbalanced distribution of the health workforce, which may be influenced by the specialty career preferences of medical students. In spite of this, there is inadequate literature concerning the factors influencing specialty career preferences. We aimed to determine the specialty career preferences and the factors influencing the preferences among fifth year medical students in the School of Medicine, Makerere University College of Health Sciences (MakCHS).

**Methods:**

A sequential explanatory mixed methods study design with a descriptive cross-sectional study followed by a qualitative study was used. A total of 135 final year medical students in MakCHS were recruited using consecutive sampling. Self-administered questionnaires and three focus group discussions were conducted. Quantitative data was analysed in STATA version 13 (StataCorp, College Station, Tx, USA) using descriptive statistics, chi-square tests and logistic regression. Qualitative data was analysed in NVIVO version 12 (QRS International, Cambridge, MA) using content analysis.

**Results:**

Of 135 students 91 (67.4%) were male and their median age was 24 years (IQR: 24, 26). As a first choice, the most preferred specialty career was obstetrics and gynecology (34/135, 25.2%), followed by surgery (27/135, 20.0%), pediatrics (18/135, 13.3%) and internal medicine (17/135, 12.6%). Non-established specialties such as anesthesia and Ear Nose and Throat (ENT) were not selected as a first choice by any student. Female students had 63% less odds of selecting surgical related specialties compared to males (aOR = 0.37, 95%CI: 0.17–0.84)**.** The focus group discussions highlighted controlled lifestyle, assurance of a good life through better financial remuneration and inspirational specialists as facilitators for specialty preference. Bad experience during the clinical rotations, lack of career guidance plus perceived poor and miserable specialists were highlighted as barriers to specialty preference.

**Conclusion:**

Obstetrics and Gynecology, Surgery, Pediatrics and Internal Medicine are well-established disciplines, which were dominantly preferred. Females were less likely to select surgical disciplines as a career choice. Therefore, there is a need to implement or establish career guidance and mentorship programs to attract students to the neglected disciplines.

## Background

The World Health Organization (WHO) recommends a doctor to patient ratio of 2.3 doctors per 1000 people for adequate health care services [1]. In spite of this, Africa has a doctor patient ratio at 0.25:1000 which is low when compared to the WHO standard [2]. This has resulted into uneven distribution of the health workforce within the various medical specialties [2]. In Uganda, this uneven distribution of health workers can be demonstrated by the number of specialist doctors registered by the Uganda Medical and Dental Practitioners Council. Among the 2012 registered specialists by 2019, 386 (19.2%) were public health specialists, 376(18.7%) were Pediatricians, 287 (14.3%) were Obstetrics and gynecology specialists, 230 (11.4%) were Internal medicine specialists and 185 (9.2%) were General surgeons [3]. There are specialties that had less than 20 (1%) specialists including ENT, Emergency medicine, and Nutrition.

It has been reported that the uneven distribution of the health workforce may occur as a result of specialty career preferences of medical students and their eventual choices of professional training [2, 4]. Studies conducted in Asia showed that students usually preferred already established clinical specialties like Pediatrics, General Surgery, Internal Medicine and Obstetrics and Gynecology [5, 6]. This pattern of preference can also be observed among medical graduates in Uganda as reflected in the admissions for post graduate programs in the past 10 years [7]. This imbalance in the health workforce may potentially lead to inadequate services offered in health care delivery [4] . Therefore, there is a need to attract medical students to the specialties with inadequate number of personnel so as to improve overall health care [2, 8, 9].

The medical students’ preferences for a particular clinical specialty is likely to be influenced by both individual and contextual factors [10]. An understanding of the career preferences of medical undergraduates can perhaps help to provide important information that can be utilized when planning educational programs. In addition, it can help to set priorities so as to foster the provision of adequate health care through training a wide range of specialists [10]. However, there is insufficient literature concerning the factors that influence specialty career preferences of medical students not only in Uganda, but also in many resource-constrained countries. Therefore, this study was designed to determine specialty career preferences and the factors that influence these preferences among final year medical students at Makerere University College of Health Sciences (MaKCHS). Our study could provide important information to be used in setting priorities and planning educational programs so as to attract students to the less preferred specialties.

## Methods

This study employed a sequential explanatory mixed study design (quantitative followed by qualitative). A quantitative cross-sectional study was conducted to determine the specialty career preferences. The qualitative study was done after the quantitative study so as to further explore the factors influencing the preferences. The study was conducted among final year medical students who had given written informed consent. Medical students in their final year were selected because they had adequate experience through medical school with satisfactory clinical experience. These students were also on the verge of entering the professional practice and had perhaps started reflecting upon the issue of specialty career preferences.

### Quantitative component of the study

Regarding sample size determination, the entire population of 178 final year medical students was considered for the study, therefore, there was no need of sampling. Permission was obtained to access the list of registered fifth year medical students during the study period from the Principal of the College of Health Sciences, on submission of the ethical approval letter. The principal investigator (PI) worked with research assistants to recruit the students for the study using convinience sampling. There was communication made regarding the study to all the fifth year medical students through messages, phone calls and verbal announcements. This was done in the various class groups with the help of class representatives who also provided the students’ contacts. The students who were interested in participating in the study were mobilized into a lecture room where the study was explained to them and they were given individual informed consent forms to read and sign. Those who did not give consent were given an opportunity to opt out of the study. Those who gave informed consent were given a self-administered structured questionnaire to collect data from them regarding their specialty career preferences. The students were given 30 min to complete the questionnaires and hand them back to the research assistants.

The structured questionnaire included questions on the demographics of the participants, their specialty career preferences (first, second and third choices), willingness to practice medicine and career guidance. The data was entered using Epidata and analysis done using STATA Version 13 (StataCorp, College Station, Tx, USA). Specialty career preferences were presented using frequencies, percentages and bar graphs. The first choice for specialty career preference was reported and compared across the socio-demographic variables using chi-square /Fisher’s exact tests in order to determine if there was an effect of these variables on the preferences. Thereafter, odds of selection of surgical versus nonsurgical discipline for first choice were obtained using multivariable logistic regression.

### Qualitative study component of the study

Following the quantitative arm of the study, the qualitative arm was carried out to explore the factors influencing specialty career preferences of medical students. The design used in this study was based on the phenomenological strategy of inquiry that is best suited to explore the experiences of students [11]. There were three Focus Group Discussions (FGDs) that were conducted in total. Two of these were homogenous groups (male and female) that comprised of students who had joined medical school at University direct from high school. Each of these two FGDs had 10 participants. The third FGD comprised of 11 students (6 males and 5 females) aged 25 years and above who had joined medical school after completion of a diploma or a medical related bachelor’s degree. Participants for these FGDs were selected using purposive sampling and taken to a quiet place on the college premises where the study objectives and procedure were explained to them face-to-face. Written informed consent was obtained from the participants before commencement of the discussions. Permission to record the discussions using both audio and written means was also obtained from the participants. The discussions were conducted by JK and DM who have expertise in qualitative methods and they lasted approximately 1 h each. The discussions were conducted using pretested FGD guides that had semi-structured questions. These questions aimed at obtaining data on the demographics of the participants, their experiences in the different specialty rotations, their most preferred specialty and the reasons that influenced this preference. There were no other FGDs conducted since data saturation had been attained by the third discussion. The audio-recorded responses were transcribed and analysed using Nvivo version 12 (QRS International, Cambridge, MA). Content analysis was used in analysis where The transcripts of the FGDs were reread a couple of times by JK and DM so as to get familiar with the content. Thereafter, coding was done by grouping words, sentences or paragraphs that relayed a similar message and then condensing them to form codes. The codes were aggregated to form categories which were further analysed to form subthemes and themes by searching for repetitive concepts, metaphors and basing on content areas chosen before data collection. Thereafter JK used the major themes and subthemes to provide descriptive and explanatory summaries of the qualitative data.,

Permission to conduct this study was granted by the Research and Ethics Committee, School of Medicine, Makerere University (Protocol No: #REC REF 2019–045.). All participants provided written informed consent and anonymity of participants including their responses  was ensured.

## Results

**Fig. 1:**
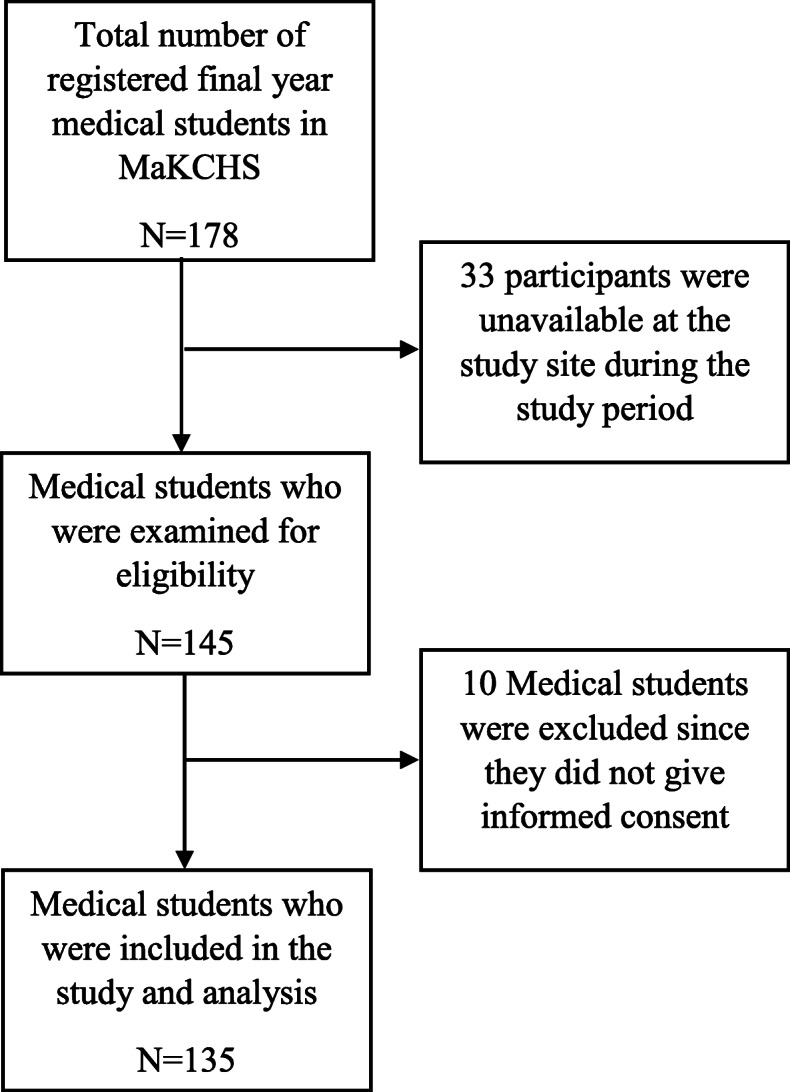
The study flow. This is a figure showing how a sample was obtained for the study. There were 179 medical students registered for the 2018/2019 academic year. Among these 33 were not available at the university during the study period therefore 145 students were screened for eligibility. Out of the 145, 10 did not consent to participate leaving a total of 135 fifth year medical students recruited into the study

The study was conducted between April and May 2019. There were 178 students registered final year medical students. However, 33 of these were not available at the university during the study period. Therefore, 145 medical students were eventually recruited into the study. The recruitment of study participants is shown in Fig. 1. Out of these, 135 students returned their questionnaires giving a response rate of 93%.

### Sociodemographic characteristics of fifth year medical students at MakCHS

Out of the 135 medical students that participated in the study, 91(67.4%) were males and 44(32.6%) were females, giving a male to female ratio of 2:1. Their ages ranged between 21 years and 47 years, with a median of 24 (IQR: 24, 26). The sociodemographic characteristics of the students are shown in Tables 1 and 2 in Appendix.

### Specialty career preferences among final year medical students at MakCHS

**Fig. 2:**
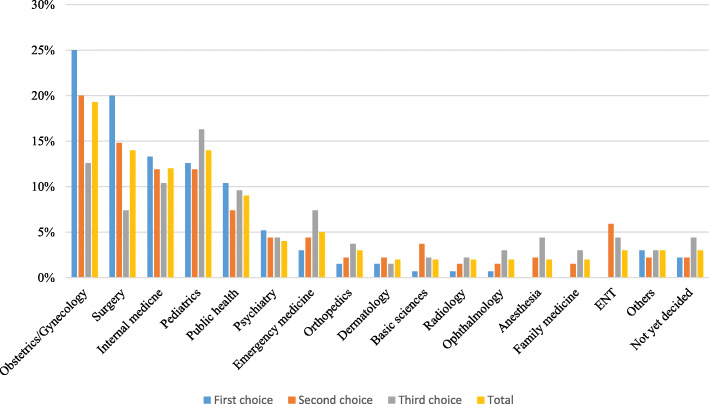
The distribution of specialty career preferences divided into first second and third choice among fifth year medical students in MaKCHS during the 2018/2019 academic year. This figures shows the details of the proportions of students that selected various careers as first second or third choice. Each specialty has four bars, the first one is blue and indicates first choice, second is orange indicating second choice, then gray indicating third choice and yellow indicating the overall total. Obstetrics and gynecology was the most commonly selected specialty overall with almost 20% of the students selecting it. About 25% of the medical students selected this specialty as the first choice. Careers like anesthesia, family medicine and ENT were not selected as first choice for any of the students

Obstetrics and Gynecology was the most commonly preferred career as a first choice, with 34 (25.2%) students selecting it; followed by careers in Surgery, Internal Medicine and Pediatrics respectively as represented in Fig. 2. Careers in Family Medicine, Anesthesia and ENT were not selected by any student. The “others” first choice category consisted of Business, Clinical epidemiology and Biostatistics, Forensic Medicine and voluntary medical work.

Obstetrics and Gynecology was still the most preferred second choice with 27 (20%) students selecting it. The “others” second choice category consisted of Nano medicine/ nuclear medicine and political science/public administration.

Pediatrics was the most preferred third choice with 22 (16.3%) students selecting it. The “others” third choice category consisted of Biogenetics science, health informatics and sports medicine. The details of the various choices as selected by the students are illustrated in Fig. 2.

### Comparison of first choice specialty preference across the sociodemographic characteristics

The distribution of the first choice specialty was compared across the various categories of the sociodemographic characteristics using the chi-square test. The test showed that there was a significant relationship between the first choice preference and sex (*p* = 0.04), marital status (*p* < 0.01), having children (*p* < 0.01) and sponsorship (*p* = 0.04) (Table 3 in Appendix). On adjustment using multivariate logistic regression, sex was the only factor with a statistically significant odds ratio (OR) (OR = 0.37; 95%CI (0.17–0.84); *p* = 0.02). Therefore, the female medical students are 63% less likely to select surgical disciplines as a specialty career preference compared to the male medical students. The results for the odds are shown in Table 4 in Appendix.

**Fig. 3:**
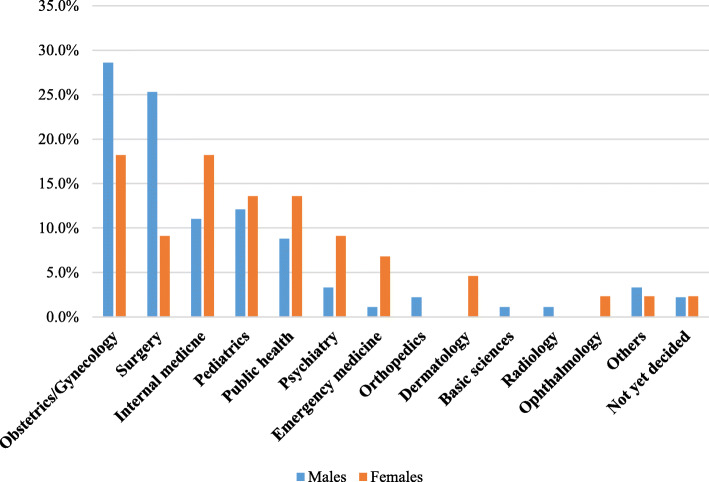
The comparison of first choice career preferences by sex among fifth year medical students in MaKCHS during the 2018/2019 academic year. Sex was shown to have a significant association with specialty career preferences among the medical students. Therefore, this figure was used to demonstrate the difference in selection of careers between male medical students and female medical students. With each specialty there is a blue bar representing the proportion of males and orange showing the proportion of females that selected that career. There was a higher proportion of male medical students that selected obstetrics and gynecology and surgery compared to the female medical students. There was a higher proportion of females than males that selected non-surgical careers including internal medicine, pediatrics, public health, psychiatry and emergency medicine. Orthopedics, basic sciences and radiology were selected by only male medical students. Dermatology and Ophthalmology were selected by only female medical students. An equal proportion of the male and female medical students were undecided about their first choice of specialty career

The female medical students were less likely to select surgical disciplines like Obstetrics and Gynecology and General Surgery when compared to their male counterparts (Fig. 3).

### Factors influencing specialty career preferences of the medical students

Three FGDs were conducted in May 2019 among 15 males and 16 females whose ages ranged from 21 to 35. The students were well distributed in terms of places of origin and different backgrounds with one foreign students included. Most of the students had a specialty career preference in mind at the time of the study.

Analysis of these FGDs revealed two major themes which are summarized in Table 5 in Appendix with the key representative quotes from the students.

#### Theme 1: facilitators of specialty career preference

The students selected various careers and based their preferences on the following influencing factors.

1. *Presence of role models and mentors:*

A key facilitator to selecting a specialty career preference among medical students was presence of role models and mentors where?? As faculty? In clinical practice? as illustrated by a participant “*… She is not only a surgeon but also a mother. The way she takes you, she respects you even though you are an undergraduate. She is really good and really motivates you to think about more of surgery”* (Mixed FGD). Presence of mentors is also very crucial since these mentors offer guidance for the student as they build their careers.

2. *Organization in the department*

Most of the students in the discussions mentioned that an organized department was a sure facilitator for selection of a career preference. A student highlighted the importance of the organization of the discipline *“When you look at those two departments, they have a good streamlined program. They try to bring out their lectures very well, associate with students very well, and evaluate well when compared to other departments.”* (Mature entrant FGD)

3. *Controlled lifestyle:*

Ability to have a controlled lifestyle also motivated students to select certain specialties. This controlled lifestyle means that one is able to balance between work, family, leisure and any other activities,*“I feel like psychiatrists have better working hours, surgeons are called in at 2am because a patient has gone into cardiac arrest or a mother has gone into labour.”* (Male FDG).

4. Media

Certain students selected certain specialities due to influence from media either through books, television series or movies. There was a student in the mixed discussion group who mentioned “*I was inspired to do internal medicine by Dr. House from the medical drama series “House”. I basically fell in love with the way they would come up with complex diagnoses by thinking through and doing various tests*.” (Male, Mixed FGD).

Other facilitators highlighted in the discussions included a positive or good experience during clinical rotation, attitudes of the specialists in the discipline, assurance of a good life due to better financial remuneration, availability of job opportunities, family influence, and peer influence.

#### Theme 2: barriers to selection of specialty career preference

1. *Perceived low financial status of the specialists*

The students’ perception of the specialists to have a low financial status is a major barrier to the selection of certain specialties *“… if I look at certain specialty and to me all the doctors look poor and miserable, the residents are broke and suffering … I am not inspired by all that knowledge they have.”* (Female FGD). Therefore most students tend to avoid specialties where it is assumed that there is a weight of challenges and financial difficulties attached.

The prolonged amount of time spent in training within a certain specialty compared to others is also a barrier. This was especially among female students who had a concern about settling and starting a family before they are too old as shown in this quote *“… well we have a biological clock to worry about … I will always go for shorter courses mainly in research with more money.”* (Mixed FGD).

2. *Uncontrollable lifestyle attached to a career*

The uncontrollable lifestyle of a specialty career is also a barrier in selection of certain careers since students want to be able to balance between life and work. There are some careers that are very demanding (especially surgical-related careers) and may not allow time for any other activities or for family *“Given the kind of mother I would like to be I may not be able to do emergency medicine; maybe because I have not got guidance from a successful emergency medicine specialist. But from what we see on TV, clearly you cannot take care of your kids and be an emergency medicine specialist.”* (Female FGD).

3. *Inadequate exposure during clinical rotations*

Inadequate exposure to a specialty during the clinical years may also be a barrier since a student may not able to appreciate the various aspects of the discipline “*I think the challenge there is the small duration we spend or the inadequate duration we are given. “We have to learn and understand because we are going to manage human beings.”* (Mixed FGD).

Other barriers to selection of specialty career preference include: Inadequate training methods used in delivering the curriculum, perceived high risk for litigation cases, strict assessments given in a rotation, lack of career guidance and students’ negative experience during clinical rotations.

## Discussion

The purpose of this study was to determine the specialty career preferences among final year medical students at MaKCHS as well as to explore factors that influence the career preferences.

From the findings of this study, obstetrics and gynecology was the most preferred specialty followed by surgery, pediatrics and internal medicine. This pattern of preference is similar to the pattern of distribution of specialists among the medical personnel in Uganda registered by the UMDPC by 2019 [3]. These specialty careers have been shown to dominate students’ choices within most medical schools globally [12]. The findings are similar to those reported in studies done at The University of The Gambia (UTG) medical school and at four medical schools within Pakistani [13, 14]. Pakistani and Gambia are also low resource countries just like Uganda. However, the results differ slightly from the findings reported in a study done in Yamaguchi University School of Medicine found in Japan where internal medicine was the most preferred specialty career with 52.7% (260/493) of the students selecting it [15].

Obstetrics and gynecology was the most preferred specialty career overall across the three choices selected by the students which can be explained by various factors highlighted during the FDGs. The obstetrics and gynecology department was perceived by the students to be very organized with inspirational and motivating specialists who were willing to work with students.

On the other hand, surgery and internal medicine were selected by students due to the perceived financial rewards attached to these careers that lead to assurance of living a good life. These careers could have also been selected by students who are driven by passion [16] or are motivated by the prestige attached to these specialties [17]. The preference for surgery can also be influenced by presence of role models as shown in a study done at Jordan University of Science and Technology among the second, fourth and sixth year medical students [18]. These role models maybe relatives, neighbors, personal physicians, family friends, or medical school faculty members.

In spite of the fact that surgery and internal medicine were selected among the top four, some students were strongly against selecting them as a preference due to the uncontrollable lifestyle associated with them. There was also media influence in selection of internal medicine as a preferred career. Watching certain medical series on television like *House* inspired some of the students to become internal medicine specialists. *House* is an American medical drama whose main character leads a team of diagnosticians at a fictional hospital in New Jersey. Internal medicine may also be preferred due to the complex intellectual content required in the process of diagnosis [17].

There was a significant difference in the way males and females selected their specialty preferences. The most preferred specialty among male students was obstetrics and gynecology followed by surgery, pediatrics and internal medicine. The high proportion of males choosing obstetrics and gynecology is similar to what was seen in a study done at Ahmadu Bello University medical school in Nigeria [19] where obstetrics and gynecology was their top surgical choice by the students. However it is different from the pattern in a study done at University of Nairobi in Kenya where most male students chose Surgery (40.0%) followed by Internal Medicine (15.0%) [17].

Among female respondents, both obstetrics and gynecology and internal medicine tied for the preferred first choice of specialty, followed by pediatrics which is similar to a study done in Jordan University of Science and Technology [18]. However, this is very different from that carried out in University of Nairobi where females chose Pediatrics as their first choice (40.0%) followed by Surgery (28.0%) [17].  It also differs from a study done in Bangladesh at Bangladesh Medical College and Uttara Adhunik Medical College where the internal medicine and pediatrics were top choices among females [10].

The pattern of males being drawn to surgical disciplines and the females being drawn to medical disciplines can be explained by the qualitative findings where most females expressed that they wanted a controllable lifestyle to be able to balance work and other areas of life like family responsibility while the males did not mind having an uncontrolled lifestyle as long as the career came with prestige. The surgical disciplines tend to be time consuming and lead to an uncontrolled lifestyle since unpredictable emergencies can come up.

Some specialties were not selected by the students as first choice such as anesthesia, ENT and family medicine. The students seemed to allude to the fact that they had limited exposure to some of these disciplines, for instance 1 week of rotation in anesthesia. This finding is in resonance with findings in a study conducted in Jordan University of Science and Technology where students did not select careers like anesthesia, ENT or family medicine due to the perception that there are diminished career opportunities [18]. However, these findings are contrasted by findings in a study conducted in University of British Columbia where family medicine was the most preferred specialty career due ready availability of jobs for these specialists [16]. In Uganda, medical students during undergraduate training have limited exposure to the family medicine and there are also limited job opportunities within the Uganda Health service Commission [20]. Therefore, students are probably not motivated enough to select family medicine a first choice specialty career.

There were students who were not interested in pursuing medical-related specialties but were interested in other areas like business or music. The students believed that they could be able to make more money in other career fields so as to sustain family and other financial needs. These findings are in agreement with those reported in a study carried out in University of Nairobi, Kenya where a proportion of the students selected non-medical careers including business, law and music [17].

About 2.5% of the students were not yet decided on the preferred specialty career. This proportion is low when compared to findings in a study done by Maseghe et al. at University of Nairobi where they found that 15.1% of the students had not yet identified their future specialty career path [17]. The lack of a decision could be due to lack of adequate career guidance as shown by the fact that only 51(62.2%) of the students had ever received any form of career guidance. Therefore, there is a need for career guidance and mentorship programs as expressed by the students in the FGDs.

### Strengths of the study

This study employed a mixed methods design where the qualitative component was used to complement and explain the quantitative results. This provided a more detailed explanation to the various preferences for specialty careers by the medical students.

This study addressed specialty career preferences among final-year medical students in Uganda and it yielded important findings, which can be used to plan for future, more comprehensive cohort studies following up the students from the medical school to the actual time they choose the specialties. The findings will impact the way in which institutions train their students so as to prepare them for selection of careers to avoid imbalance in the health workforce.

### Limitations

The results of this study may be viewed in the context of the following limitations. The specialty career preference was measured at one point in time yet literature suggests that the choice does not remain stable over the course of medical education and medical practice after undergraduate study. The preferences may be refined over the internship period or the time they start practicing as licensed doctors. Some students may not have developed a preference for any specialty by the time of the study and may have given speculative information. However, this was minimized by selecting medical students in their final year who had been exposed to the various specialties during their clinical rotations.

The study was conducted in only one medical school within MakCHS therefore the results may not be generalizable to the entire country since the various universities are in different settings. Inspite of this, Makerere University has the biggest intake of medical students compared to other public universities.

## Conclusion

The most preferred specialty career among the fifth year medical students at MakCHS was obstetrics and gynecology. The other top careers selected include pediatrics, surgery and internal medicine. There were career options that were not selected as a first choice including anesthesia and ENT due to limited exposure.

The factors influencing the specialty career preferences include gender where males preferred the surgical disciples compared to females who preferred the medical disciplines that could allow them to balance work and family life. Other factors facilitating career preferences included controlled lifestyle preference, assurance of good life and inspirational specialists. The barriers for selection of some specialty careers included bad experience in clinical rotations, lack of guidance, perceived poor and miserable specialists.

A study conducted on a larger scale including other medical schools in Uganda is required so as to determine the factors that are associated with specialty career preferences of final year medical students. There is also a need for further research in form of a follow up study among students from undergraduate through post graduate to post-education phase in order to determine the factors associated with the eventual specialty career choices.

## Data Availability

The datasets used and/or analyzed during the current study are available from the corresponding author on reasonable request.

## Appendix

**Table 1:** The socio-demographic characteristics of fifth year medical students in MakCHS during the academic year 2018/2019

Variable	Frequency (***N*** = 135)	Percentage
**Age (*****n*** **= 134)**		
21–24 years	70	52.2
≥ 25 years	64	47.8
**Sex**		
Male	91	67.4
Female	44	32.6
**Nationality**		
Ugandan	130	96.3
Non Ugandan	5	3.7
**Religion (*****n*** **= 134)**		
Anglican	41	30.6
Pentecostal	37	27.6
Catholic	37	27.6
Moslem	11	8.2
Others^1^	8	6.0
**Place of Origin (*****n*** **= 133)**		
Central Uganda	118	88.7
Western Uganda	4	3.0
Eastern Uganda	8	6.0
Northern Uganda	3	2.3
**Marital Status**		
Single	121	89.6
Married	14	10.4
**Have Children**		
No	118	87.4
Yes	17	12.6
**Sponsorship**		
Government	72	53.3
Private	63	46.7

^1^Other religions include: Hindu = 1, Seventh day Adventist = 3 and No religion = 4

Table 1 above shows some of the sociodemographic characteristics of characteristics of fifth year medical students which were age, sex, religion, nationality, place of origin, marital status, having children and sponsorship (first column). Place of origin is defined as the place where the ancestors of the participant are from and within Uganda the districts of origin are grouped into four regions including central, western, northern and eastern. The participants were asked whether they have children and this was recorded as well. Sponsorship is usually based on who was paying tuition for the student throughout the period of medical school. Government sponsorship is given to a number of students who enter medical school on merit. Those who are not sponsored by the government have to pay the tuition on their own or get private organizations to cover them which is also known as Private sponsorship.

**Table 2:** The parents’ level of education and career guidance among the MakCHS fifth year medical students during the 2018/2019 academic year

Variable	Frequency(n)	Percentage (%)
**Father’s level of education**		
None	4	3.0
Primary	17	12.6
Secondary	24	17.8
Tertiary	90	66.7
**Mother’s level of education**		
None	9	6.7
Primary	23	17.0
Secondary	33	24.4
Tertiary	70	51.8
**Ever received career guidance counseling**		
Yes	84	37.8
No	51	62.2
**Want a workshop on career or specialty counseling before internship**		
Yes	124	91.9
No	11	8.2

Table 2 shows the parents’ level of education which is divided into four levels. The first level is none which is basically those who have never received a formal education. The second level is Primary, third is secondary ad fourth is tertiary. The tertiary level consists of all post-secondary education including universities, colleges, technical training institutes, and vocational schools. The participants were asked if they had ever received any form of career guidance counselling and whether they were interested in a workshop. This was asked so as to assess the students’ willingness and interest in counselling. The results were presented as frequency (second column) and percentage (third column).

**Table 3:** Specialty career preferences (first choice) based on the demographic characteristics

Variable	Overall(***N*** = 135)	Obs & Gyn(***n*** = 34)	Surgery(***n*** = 27)	Internal medicine(***n*** = 18)	Pediatrics(***n*** = 17)	Others(***n*** = 39)	Chi-square/Fisher’s value
**Age (*****n*** **= 134)**							
21–24 years	70	12 (17.1)	15 (21.4)	10 (14.3)	9 (12.9)	24 (34.3)	0.22
≥ 25 years	64	22 (34.4)	12 (18.8)	7 (10.9)	8 (12.5)	15 (23.4)	
**Sex**							
Male	91	26 (28.6)	23 (25.3)	10 (11.0)	11 (12.1)	21 (23.1)	0.04*****
Female	44	8 (18.2)	4 (9.1)	8 (18.2)	6 (13.6)	18 (40.9)	
**Religion (*****n*** **= 134)**							
Anglican	41	11 (26.8)	7 (17.1)	6 (14.6)	6 (14.6)	11 (26.8)	0.57
Pentecostal	37	7 (18.9)	8 (21.6)	6 (16.2)	5 (13.5)	11 (29.7)	
Catholic	37	10 (27.0)	7 (18.9)	6 (16.2)	3 (8.1)	11 (29.7)	
Moslem	11	4 (36.4)	–	–	3 (27.3)	4 (36.4)	
Others^a^	8	2 (25.0)	4 (50.0)	–	–	2 (25.0)	
**Place of Origin (*****n*** **= 133)**							
Central Uganda	118	29 (24.6)	23 (19.5)	17 (14.4)	14 (11.9)	35 (29.7)	0.30
Western Uganda	4	2 (50.0)	–	1 (25.0)	1 (25.0)	–	
Eastern Uganda	8	2 (25.0)	–	–	2 (25.0)	4 (50.0)	
Northern Uganda	3	1 (33.3)	2 (66.7)	–	–	–	
**Marital Status**							
Single	121	24 (19.8)	25 (20.7)	18 (14.9)	17 (14.1)	37 (30.6)	< 0.01*****
Married	14	10 (71.4)	2 (14.3)	–	–	2 (14.3)	
**Have Children**							
No	118	24 (20.3)	23 (19.5)	18 (15.3)	17 (14.4)	36 (30.5)	< 0.01*****
Yes	17	10 (58.8)	4 (23.5)	–	–	3 (17.7)	
**Sponsorship**							
Government	72	15 (20.8)	16 (22.2)	11 (15.3)	14 (19.4)	16 (22.2)	0.04*****
Private	63	19 (30.2)	11 (17.5)	7 (11.1)	3 (4.8)	23 (36.5)	

^a^Other religions include: Hindu = 1, Seventh day Adventist = 3 and No religion = 4

*This symbol denotes the p-values that are below 0.2 for variables that were considered for further analysis

Table 3 shows the distribution of First choice specialty preferences among the various sociodemographic characteristics of the fifth year medical students. The students were asked to select three preferences and rate them as first second and third choice. The most commonly selected specialty as first choice was obstetrics and gynecology followed by surgery, internal medicine and pediatrics. About 39 out of 135 selected other specialties including public health, psychiatry, emergency medicine, orthopedics, dermatology, basic sciences, radiology, ophthalmology, Business, Clinical epidemiology and Biostatistics, Forensic Medicine and voluntary medical work. Among these was also a proportion of students who were not yet decided. These were then divided in to five groups consisting of Obstetrics and gynecology, Surgery, Internal Medicine, Pediatrics and others. The chisquare test was used to determine if the sociodemographic characteristics had a relationship with preference of career specialties. This test was used to compare the selection of careers across the various categories of the sociodemographic characteristics. The following variables were found to have a significant relationship with first specialty career preference: Sex (*p* = 0.04), marital status (*p* < 0.01), having children (*p* < 0.01) and sponsorship (*p* = 0.04).

**Table 4:** The odds of selecting a surgical discipline (surgery and obstetrics/gynecology) as a first choice preference among the fifth year medical students in MakCHS

Variable	Specialty career preference N(%)		Adjusted OR	95% CI	***p*** value
	Non-surgical specialty	Surgical specialty			
**Sex**					
Male	42 (46.15)	49 (53.85)	1		
Female	32 (72.73)	12 (27.27)	0.37	0.17–0.84	**0.02***
**Marital status**					
Single	72 (59.50)	49 (40.50)	1		
Married	2 (14.29)	12 (85.71)	3.73	0.58–24.00	0.17
**Having Children**					
No	71 (60.17)	47 (39.83)	1		
Yes	3 (17.65)	14 (82.35)	2.87	0.58–14.08	0.2

*This symbol denotes the *p*-values that are significant at the level of α=0.05

The factors that had a relationship with selection of first choice specialty were considered for further analysis using multiple logistic regression. This was done so as to determine the odds of selection of a surgical specialty (obstetrics & gynecology, surgery) vs a non-surgical specialty. The table shows that sex had a significant odds ratio (OR) with 63% less likelihood for female medical students to select a surgical speciality when compared with male medical students (OR = 0.37; 95%CI (0.17–0.84); *p* = 0.02)

**Table 5:** Representative quotations of major themes identified from FGDs among the fifth year medical students at MakCHS

Major theme	Sub theme	Representative quote
**Facilitators for students’ specialty career preferences**	1. Controlled lifestyle preference	*“I feel like psychiatrists have better working hours, surgeons are called in at 2 am because a patient has gone into cardiac arrest or a mother has gone into labour.”(Female FGD)*
	2. Assurance of a good life	*“I think all of us would want to go to those specialties, even the trivial ones. As long as a good life is guaranteed we would like to serve.” (Male FGD)*
	3. Inspirational specialists	*“Dr. Y*^a^ *makes you feel like you are on the same level with her. If you do not know the answer Y says “Let us google”. Dr Y does not make you feel like you are stupid or so inefficient...”(Female FGD)*
**Barriers to students’ specialty career preferences**	1. Inadequate exposure	“*I think the challenge there is the duration we spend or the duration we are given. You cannot tell me to go to anesthesia for one week yet it has 5 credit units. I have to learn and understand because we are going to manage human beings.”* (Mature entrant FGD)
	2. Lack of career guidance	*“So yes I have never thought of it as a career choice because no one ever put it forth and showed us how good it can be.”* (Male FGD).
	3. Perceived low financial status of the specialists	*“… I could not consider the specialty because according to me, those people do not look happy. The way I see them, I would not want to be like them.” (Males FDG)*

^a^Name withheld for confidentiality reasons

Table 5 shows the major themes, subthemes and their representative quotes. The themes were derived from transcripts obtained from the focus group discussions. The transcripts were analysed using content analysis then sub-themes were obtained. Thereafter the subthemes were categorized into two major themes including facilitators for students’ specialty career preferences and barriers to students’ specialty career preferences. These themes showed the factors that were influencing the Specialty career preferences of medical students.

## Abbreviations

ENT: Ear Nose and Throat
FDG: Focus Group Discussion
DRGT: Directorate of research and graduate training
MakCHS: Makerere University College of Health Sciences
MOH: Ministry of Health
PI: Principal Investigator
UMDC: Uganda Medical and Dental Council
WHO: World Health Organisation
